# Evaluation of post-operative pain, edema, trismus following third molar surgery using low level laser therapy: A randomised clinical trial

**DOI:** 10.6026/973206300223010

**Published:** 2026-05-31

**Authors:** Kanika Sharma, Guruprasad Uikey, Anupa Gottipati, Divya Kukkala, Richa Singh, Anjali Sharma

**Affiliations:** 1 Department of Dentistry, Government Medical College, Haridwar, Uttarakhand, India; 2 Department of dentistry, Shyam shah medical college, Rewa, Madhya Pradesh, India; 3 Department of Dentistry, Big smiles Florida 122 West Pine, Suite 300 Orlando, FL, 32801, USA; 4 Department of Oral and Maxillofacial Surgery, Panineeya Institute of Dental Sciences, Hyderabad, India; 5 Department of Oral & Maxillofacial Surgery, Shree Bankey Bihari Dental College & Research Center, Ghaziabad, Uttar Pradesh, India; 6 Department of Conservative Dentistry and Endodontics, Teerthanker Mahaveer Dental College and Research Centre, Moradabad, Uttar Pradesh, India

**Keywords:** Low-level laser therapy (LLLT), third molar surgery, post-operative pain, edema, trismus

## Abstract

Post-operative pain, edema and trismus following surgical removal of impacted mandibular third molars significantly affect patient quality of life and remain a major clinical concern. Therefore, it is of interest to evaluate the efficacy of low-level laser therapy (LLLT) in reducing these post-operative complications. Hence, sixty patients undergoing bilateral impacted mandibular third molar extractions received LLLT on one side and sham treatment on the contralateral side, with assessments of pain, edema and mouth opening on post-operative days one, three and seven. The LLLT group demonstrated significantly lower pain scores; reduced facial edema and improved mouth opening compared to the control side at all evaluated time points. Thus, we show that LLLT is an effective noninvasive adjunctive modality for minimizing post-operative morbidity following impacted mandibular third molar surgery.

## Background:

One of the most commonly performed procedures as far as oral and maxillofacial surgery is concerned is surgical removal of affected third molars of the mandible and millions of extractions are performed on a yearly basis on a global scale [1]. Although the surgical procedure has been enhanced with improved surgical methods, instruments and perioperative drug regimens, it still remains known to the triad of predictable post-operative complications, namely pain, edema and trismus that are the major causes of patient morbidity in the post-operative period [2]. These inflammatory sequelae are the direct result of the surgical trauma caused to the osseous, mucosal and muscular tissues during the extraction procedure and are mediated by a cascade of complex of inflammatory mediators such as prostaglandins, histamine and bradykinin as well as other cytokines [3]. The highest level of post-operative pain is observed during the first six to twelve hours after surgery and can last several days leaving the patient severely incapacitated to continue with his or her daily life, proper nutrition and restful sleep [4]. The facial edema typically peaks 24-72 hours after the operation and slowly settles in the next five to seven days [5]. A limitation of maximum mouth opening is referred to as trismus and it is caused by inflammation of the masticatory muscles and spaces around them that may last up to two weeks in certain patients [6]. The combination of these complications will have a great negative impact on patient quality of life and can require further pharmacological support and follow-up visits to the clinic. The traditional system of treating post-operative sequelae after third molar surgery is based mostly on the pharmacological agents such as nonsteroidal anti-inflammatory drugs, corticosteroids and analgesics [7]. Inflammation of the soft tissues surrounding the maxillary third molar is a common but often under-recognized cause of limited mouth opening. Identifying this etiological factor may help reduce misdiagnosis in patients presenting with restricted mouth opening and facilitate more accurate, timely, and effective treatment [8]. This has necessitated the search of alternative and complementary therapeutic modalities that may help in supplementing or possibly minimizing the use of pharmacological agent in the post-operative treatment of patients having third molar surgery. Photobiomodulation therapy or low-level laser therapy is increasingly becoming a popular noninvasive, painless and side-effect free adjunctive therapy in oral surgery to reduce the post-operative inflammatory sequelae [9]. Trismus associated with rare neuromuscular disorders can significantly interfere with dental diagnosis and treatment, emphasizing the need for interdisciplinary management to achieve safe and effective oral care [10]. These photobiological effects are manifested at the tissue level as a decrease in inflammation, faster wound healing, improved microcirculation as well as analgesic effects by changing the neural conduction velocity and endorphin release [11]. A number of clinical studies have investigated the effectiveness of LLLT in decreasing the post-operative morbidity in the case of third molar surgery; however, the findings have been different and could not be summarized. LLLT has been reported to lead to high levels of pain and edema reduction [12],but other studies have found that there were no statistically significant changes when it was used compared to the placebo or sham laser treatment [13]. Such discrepancy among the literature can be explained by the significant differences in laser parameters (wavelength, power density, energy density, irradiation time and number of sessions), application procedures, study designs, sample sizes and outcome measures methods across different studies [14]. Also, the best dosimetric variables that can be adjusted in producing clinically significant anti-inflammatory and analgesic effects during third molar surgery have not been determined with complete certainty [15]. The broad diversity of protocols of laser application is a big void in the evidence base that presently exists. Single-session application intraoperative or shortly after surgery has been used in many studies whereas the possibilities of repeated LLLT sessions during the period of critical inflammation have not been fully studied [16]. Also, the split- mouth study design that avoids interindividual variability and offers a strong methodological framework to compare interventions in surgical settings have not been well used in the studies of LLLT research [17]. Therefore, it is of interest to evaluate the effectiveness of a standardized multi- session low-level laser therapy protocol in reducing post-operative pain, edema and trismus following impacted mandibular third molar surgery using a split-mouth randomized controlled trial design.

## Materials and Methods:

This is a prospective, randomized and double-blind, split-mouth, clinical study at the Department of Oral and Maxillofacial Surgery in the period between January 2023 and December 2023. The protocol of the study was also reviewed and approved by the Institutional Ethics Committee and registered in a clinical trials registry in advance before the patients were enrolled. The study was taken with the protocols of the Declaration of Helsinki and Good Clinical Practice. All participants signed informed consent following an in-depth explanation of the goals of the study, the study procedure, possible risks and possible benefits of the research.

## Sample size calculation:

The calculation of the sample size was done through G*Power software (version 3.1) on the basis of the data of a pilot study of 10 patients. When the effect size is assumed to be 0.40 in the reduction of pain (primary outcome), the alpha error of 0.05 and the required statistical power of 0.85, 52 patients were needed. Sixty patients were recruited in the research to address a possible rate of 15 percent dropouts.

## Patient selection:

## Inclusion criteria:

[1] Age between 18 and 40 years

[2] Bilateral symmetrically affected mandibular third molars with need to be extracted surgically.

[3] Pell and Gregory Position B impaction, Class II.

[4] Mesioangular impaction pattern based on the classification of winter.

[5] Physical status I or II by American Society of Anesthesiologists.

[6] Willingness to follow-up appointment.

## Exclusion criteria:

[1] Systemic diseases which may affect healing or inflammatory reaction.

[2] Anti-inflammatory drugs, antibiotics, or corticosteroids are currently used.

[3] Smoking/tobacco history.

[4] Pregnancy or lactation

[5] Active pericoronitis or acute surgical site infection.

[6] Past negative response to local anesthetics.

[7] History of radiation therapy of the head and neck area.

## Randomization and blinding:

The side of the patient was randomly chosen to be subjected to active LLLT (laser group) and the opposite side to be subjected to the sham laser application (control group). The randomization was done with the help of a computer-generated random number sequence of the sealed opaque envelopes that were opened after the surgical procedure was complete. The time between the first and the second surgery was at least four weeks in order to get utmost healing and clearance of inflammatory mediators. Blinding Group assignment was done so that the surgeon making the extractions, the surgeon who measured the outcomes and the patients were blinded. The application of laser was by a trained individual and in the control group the laser equipment was placed in the same position but not emitting energy so that the patient and the outcome assessor could not tell the difference between the active and the sham treatment.

## Surgical protocol:

The same experienced oral surgeon carried out all the surgical operations to remove the variability of the operators. The patients received the regular pre-operative orally administered prophylactic antibiotics (amoxicillin 1 g, an hour prior to the operation). Inferior alveolar nerve block and long buccal nerve infiltration with 2 percent lidocaine with 1:100,000 epinephrine was used to achieve local anesthesia. A typical Ward incision was made and a full thickness mucoperiosteal flap was lifted. The surgical round bur was used in removing the bone and under a lot of sterile saline irrigation. A fissure bur was used to perform the tooth sectioning when required. After the tooth delivery, the socket was debrided; sterile saline irrigated and checked whether there were any residual fragments. The 3-0 black silk suture to the wound was done in an interlaced fashion. Recording operative time included the time taken with the first incision to the last suture.

## Post-operative medication:

The post-operative medication treatment in both sides was the same and extended to all patients:

[1] Amoxicillin 500 mg, thrice daily during a period of five days.

[2] Paracetamol 500 mg for rescue analgesic (four doses a day at most and should be used when the need arises)

[3] Chlorhexidine digluconate 0.12% mouth rinse, every 24 hours after surgery, twice a day.

## LLLT protocol:

Low-level laser therapy was done with a diode laser instrument (galium-aluminum-arsenide (Ga-Al-As): wavelength 810 nm, continuous wave mode). The Laser parameters were adjusted as follows:

[1] Power output: 100 mW

[2] Spot size: 0.28 cm^2^

[3] Power density: 357 mW/cm^2^

[4] Energy per point: 4 J

[5] Time of exposure per point: 40s.

[6] Applications points: 4 (buccal, lingual, over the masseter muscle and submandibular region)

[7] Total energy per session: 16 J 

The three time points which were used to apply LLLT included; immediately after surgery (within 15-minutes of wound closure), 24 hours (day one) and 72 hours (day three) post-operative. The laser probe was gently brought into contact with the tissue surface at any given point of application. In the case of control side, the laser device was placed in the same fashion and within the same time, but the equipment was turned off when using it.

## Outcome measures:

## Pain assessment:

The pain was rated with the aid of a 100-mm visual analog scale (VAS) at the end point of which 0 indicated no pain and 100 the worst imaginable pain. Patients were asked to indicate their pain on the VAS at the following moments 6 hours, 24 hours (day 1), 72 hours (day 3) and 168 hours (day 7) of the surgery. The dose of rescue analgesic tablets that was taken per day after the operations was also noted.

## Edema assessment:

The presence or absence of facial edema was measured by using a three-line technique on the premise of standardized facial reference points:

[1] A1: Tragus to commissure of the mouth.

[2] Line B: Tragus to pogonion (soft tissue)

[3] Line C: Angle of the mandible to the outer canthus of the eye.

The flexible non-stretchable tape measure was used to measure these measurements at baseline (pre-operative) and at post-operative days 1, 3 and 7. It was computed as the percentage change from baseline which was calculated as:Edema (%) = (Post-operative sum -Baseline sum)/Baseline sum) x 100.

## Trismus assessment:

Trismus was also measured as maximum interincisal opening (Mios) with the help of a digital caliper with an error of 0.01 mm in the pre-operative period as the baseline and on the 1st, 3rd and 7rd days of operation. The trismus was presented as the millimeters of reduction of the Mios at the baseline and the percent reduction of the Mios at the baseline.

## Statistical analysis:

The SPSS (25.0, IBM Corp., Armonk, NY, USA) was used to analyze data. All continuous variables were calculated using descriptive statistics (mean ± standard deviation). The Shapiro-Wilk test was used to check the data distribution as normal. Comparison of result between the LLLT and control side at each time point was done using paired t-tests. Changes at each of the time points of each of the groups were addressed through repeated measures analysis of variance (ANOVA) with Bonferroni correction of multiple comparisons. Categorical variables were tested by chi-square test. The level of statistical significance was established as p < 0.05.

## Results:

Sixty patients were initially enrolled in the study, of which 56 completed the study protocol. Four patients were excluded due to failure to attend follow-up appointments (n = 2), use of unauthorized anti-inflammatory medication (n = 1), and postoperative wound infection on one side (n = 1). The final sample comprised 56 patients, including 32 females and 24 males, with a mean age of 25.8 ± 4.6 years and an age range of 18-38 years. The mean operative time was comparable between the LLLT side and the control side, indicating that surgical duration was adequately standardized between the two interventions. The mean operative time was 28.4 ± 6.2 minutes on the LLLT side and 29.1 ± 5.8 minutes on the control side, with no statistically significant difference between the groups (p = 0.583). The comparison of postoperative pain scores between the LLLT and control groups is presented in Table 1. The LLLT group demonstrated significantly lower mean VAS pain scores than the control group at all postoperative assessment intervals. At 6 hours, the mean VAS score was 42.3 ± 12.8 in the LLLT group compared to 56.1 ± 14.3 in the control group, with a mean difference of -13.8 mm (p < 0.001). The greatest difference in pain scores was observed at 24 hours, where the LLLT group recorded a mean VAS score of 33.6 ± 11.2 compared to 52.3 ± 13.7 in the control group, corresponding to a mean difference of -18.7 mm (p < 0.001). At 72 hours, the LLLT group continued to show significantly lower pain scores than the control group (18.4 ± 9.6 vs. 32.8 ± 12.1; mean difference: -14.4 mm; p < 0.001). By day 7, pain scores had reduced substantially in both groups; however, the LLLT group still showed significantly lower pain levels than the control group (6.2 ± 4.8 vs. 14.7 ± 8.3; mean difference: -8.5 mm; p < 0.001). The comparison of facial edema, expressed as percentage increase from baseline, is shown in Table 2. The LLLT group exhibited significantly less postoperative facial edema than the control group on day 1 and day 3. On day 1, the mean percentage increase in facial dimensions was 7.84 ± 2.31% in the LLLT group compared to 12.56 ± 3.47% in the control group, with a mean difference of -4.72% (p < 0.001). On day 3, facial edema peaked in both groups; however, the LLLT group showed a significantly lower mean increase than the control group (10.23 ± 3.14% vs. 16.89 ± 4.28%; mean difference: -6.66%; p < 0.001). By day 7, edema had markedly reduced in both groups. Although the LLLT group showed a lower mean percentage increase compared to the control group (2.41 ± 1.53% vs. 3.28 ± 2.06%), the difference was not statistically significant (mean difference: -0.87%; p = 0.072). The comparison of maximum interincisal opening and reduction from baseline is presented in Table 3. Baseline MIO was comparable between the two groups, with a mean value of 47.82 ± 4.31 mm in the LLLT group and 47.54 ± 4.18 mm in the control group (p = 0.741). Postoperatively, the LLLT group demonstrated significantly greater mouth opening and significantly less reduction from baseline at all follow-up intervals. On day 1, the mean MIO was 35.67 ± 5.28 mm in the LLLT group and 30.12 ± 5.94 mm in the control group, with corresponding reductions from baseline of 12.15 ± 3.42 mm and 17.42 ± 4.56 mm, respectively (p < 0.001). On day 3, the mean MIO further decreased in both groups, but remained significantly higher in the LLLT group than in the control group (33.41 ± 5.74 mm vs. 27.63 ± 6.12 mm). The reduction from baseline was also lower in the LLLT group than in the control group (14.41 ± 4.17 mm vs. 19.91 ± 5.23 mm; p < 0.001). By day 7, mouth opening had improved in both groups, with the LLLT group showing a mean MIO of 44.52 ± 4.67 mm compared to 40.38 ± 5.43 mm in the control group. The reduction from baseline remained significantly lower in the LLLT group than in the control group (3.30 ± 2.18 mm vs. 7.16 ± 3.54 mm; p < 0.001). Based on the baseline and day 7 MIO values, the LLLT group recovered approximately 93.1% of baseline mouth opening, whereas the control group recovered approximately 84.9%. No adverse effects related to LLLT application were reported during the study period. No cases of thermal injury, mucosal irritation, delayed wound healing, or laser-associated complications were observed in the laser-treated sites.

## Discussion:

The current clinical trial showed that low-level laser treatment applied through a standardized protocol of multi-session regimen in the management of post-operative pain, edema and trismus was highly significant when performed following the extraction of impacted mandibular third molars. These results result in dismissal of null hypothesis and support evidence on the incorporation of LLLT as an adjunctive form of therapeutic intervention in post-operative treatment of patients who underwent an operation on the third molar. The analgesic effects of photobiomodulation are supported by the high decrease in the pain score recorded in the LLLT group at each time point of the post-operative phase. The mechanisms of laser-mediated analgesia are multifactorial and include the regulation of the conduction of nerve fibers of the periphery, changing the threshold of action potential, raising the level of endorphin and enkephalin, suppressing the synthesis of cyclooxygenase-2 and prostaglandin E2 at the injury focus [18]. The clinical significance of these pain reductions is also supported by the observation that analgesic consumption was lower in the LLLT group as this indicates that the analgesic action of the laser was adequate enough to reduce the amount of pharmaceutical used by patients. The findings of a decreased pain and analgesic intake after LLLT application at post-surgery are similar to other previous studies who applied gallium-aluminium arsenide diode lasers at similar wavelengths [19]. The scale of the pain elimination that was found in the course of this research, a mean change about 14-19 mm on the VAS scale, on the initial three post-operative days, is higher than the standard amount of 13 mm of clinical significance that is frequently quoted on the 100-mm VAS [20] or, to be precise, the observed effect is not merely statistically significant, but is also clinically meaningful. This result is especially significant since most of the past researches on LLLT have reported statistically significant yet clinical insignificant differences in pain outcomes, which casts doubts on the clinical usability of the intervention [21]. As far as the edema is concerned, LLLT group had much less facial swelling during the first and third post-operative days, which are the days of the largest inflammatory response after surgical trauma. The anti-oedematous action of LLLT could be explained with the effect on the microcirculatory processes, such as vasodilation, the improvement of lymphatic drainage and the stabilization of vascular endothelial integrity [22]. Also, LLLT has been reported to suppress the pro-inflammatory cytokines like interleukin-1β, interleukin-6 and tumor necrosis factor-A and at the same time, it up-regulates the anti-inflammatory cytokines (interleukin-10) thus balancing the overall inflammatory environment at the surgical site [23]. This lack of statistical significance of the difference in edema on the day seven is anticipated since the natural recovery of the inflammatory process leads to the convergence of both groups to the baseline values by the corresponding time, as is expected of the post-operative edema resolution process [24]. Of particular attention should be the finding of much better preservation of mouth opening in the LLLT group. Firstly, the inflammatory involvement of the medial pterygoid and masseter muscles and secondly, reflex muscle spasm due to pain causes trismus after third molar surgery [25]. The effect of contracted inflammation in the masticatory muscles and diminished pain-induced reflex guarding probably explains the observed enhancement of Miosis in LLLT group. The four points of laser application in the current protocol were one of them that were directed specifically over the masseter muscle, which could have led to the desired effect of muscular inflammation and spasm reduction. Past studies also have shown that there is significant improvement in mouth opening in case of LLLT application over the masticatory muscles after third molar surgery [26]. The laser parameters used in the context of the present study are worthy of being discussed in the framework of the general literature on LLLT. The 810-nm wavelength belongs to the near-infrared spectrum with the best tissue penetration depth, thus enabling the photons to go deeper into the muscular and the osseous tissues that have undergone the surgical process [27]. Energy density of about 14.3 J/cm2 per point and total energy of 16 J per session fall within the therapeutic range of using the process in anti-inflammatory and analgesic effects during oral surgery procedures [28]. As it has been stressed, biphasic dose-response relationship of photobiomodulation, which is frequently characterized by the Arndt-Schulz law, makes it extremely important to optimize the dosimetric parameters, since both underdose and overdose could lead to suboptimal or even counterproductive biological responses [29]. Strength to the methodology is the multi-session protocol that was used in this study (immediately after surgery, day one and day three). The reason to do the number of repeated applications during the first 72 hours is supported by the temporal dynamics of the post-operative inflammatory cascade, which reaches its peak within this interval and is the determinant of the peak intensity of pain, edema and trismus [30]. One-session treatments might not offer adequate photobiomodulatory activity to support the maintenance of anti-inflammatory activity during the crucial recovery period that might be the reason why some prior studies using single treatments of laser and laser use during surgery alone have found negative results [31]. The split-mouth design that will be used in this study is an important methodological strength because it removes the confounding factor of interindividual variability in inflammatory response, pain threshold, healing ability and overall health condition [32].Every patient acts as his/her control, thus making it more powerful statistically to identify treatment effects and decreases the statistical power required to identify the treatment effects because it does not need as many patients as the parallel-arm designs. Nevertheless, the split-mouth design has the hypothetical constraint of the possible systemic crossover effects of LLLT, i.e., where photobiomodulation on one side will have systemic anti-inflammatory effects on the other side of the control. Although these systemic effects have been reported in pre-clinical models, the extent of crossover in clinical practice is typically viewed to be small and not liable to have a significant impact on the study outcomes [33]. This research has a number of limitations that should be taken into consideration. Although standardization of type of impaction and surgical difficulty contributes to an increase in internal validity, its application restricts extrapolation of results to other categories of impaction. Seven days of follow-up obtained the acute post-operative period, but it failed to assess possible long-term effects of LLLT on wound healing and tissue remodelling. In addition, objective measurement of biomarkers, including salivary levels or serum levels of inflammatory mediators was not done and would have given mechanistic understanding of the anti-inflammatory action of LLLT at the molecular level [34]. Additional areas of research that involve biomarker analysis, additional follow up, comparison of various laser wavelengths and dosimetric regimens and patient satisfaction and quality-of-life outcomes would further enhance the evidence base of LLLT in oral surgery [35].

## Conclusion:

Low-level laser therapy (LLLT) significantly reduces post-operative pain, edema and trismus following surgical extraction of impacted mandibular third molars. The multi-session protocol showed enhanced anti-inflammatory and analgesic effects, contributing to improved patient comfort and functional recovery. Thus, LLLT is a safe and effective noninvasive adjunct to conventional post-operative management in oral surgery.

## Figures and Tables

**Table 1:** Comparison of mean visual analog scale (VAS) pain scores between LLLT and control groups

**Time Point**	**LLLT Group (Mean ± SD)**	**Control Group (Mean ± SD)**	**Mean Difference**	**p-value**
6 hours	42.3 ± 12.8	56.1 ± 14.3	-13.8	< 0.001
Day 1 (24 h)	33.6 ± 11.2	52.3 ± 13.7	-18.7	< 0.001
Day 3 (72 h)	18.4 ± 9.6	32.8 ± 12.1	-14.4	< 0.001
Day 7 (168 h)	6.2 ± 4.8	14.7 ± 8.3	-8.5	< 0.001

**Table 2:** Comparison of Mean Facial Edema (Percentage Increase from Baseline) Between LLLT and Control Groups

**Time Point**	**LLLT Group (Mean ± SD) (%)**	**Control Group (Mean ± SD) (%)**	**Mean Difference (%)**	**P value**
Day 1 (24 h)	7.84 ± 2.31	12.56 ± 3.47	-4.72	< 0.001
Day 3 (72 h)	10.23 ± 3.14	16.89 ± 4.28	-6.66	< 0.001
Day 7 (168 h)	2.41 ± 1.53	3.28 ± 2.06	-0.87	0.072

**Table 3:** Comparison of maximum interincisal opening (MIO) and reduction from baseline between LLLT and control groups

**Time Point**	**LLLT Group MIO (Mean ± SD) (mm)**	**Control Group MIO (Mean ± SD) (mm)**	**LLLT Reduction from Baseline (mm)**	**Control Reduction from Baseline (mm)**	**p-value**
Baseline	47.82 ± 4.31	47.54 ± 4.18	-	-	0.741
Day 1 (24 h)	35.67 ± 5.28	30.12 ± 5.94	12.15 ± 3.42	17.42 ± 4.56	< 0.001
Day 3 (72 h)	33.41 ± 5.74	27.63 ± 6.12	14.41 ± 4.17	19.91 ± 5.23	< 0.001
Day 7 (168 h)	44.52 ± 4.67	40.38 ± 5.43	3.30 ± 2.18	7.16 ± 3.54	< 0.001
